# Acceptability and feasibility of point-of-care CD4 testing on HIV continuum of care in low and middle income countries: a systematic review

**DOI:** 10.1186/s12913-016-1588-y

**Published:** 2016-08-02

**Authors:** Minh D. Pham, Paul A. Agius, Lorena Romero, Peter McGlynn, David Anderson, Suzanne M. Crowe, Stanley Luchters

**Affiliations:** 1 Burnet Institute, Melbourne, VIC Australia; 2 Department of Epidemiology and Preventive Medicine, Faculty of Medicine Nursing and Health Science, Monash University, Melbourne, Australia; 3 The Alfred Hospital, The Ian Potter Library, Melbourne, VIC Australia; 4 Department of Immunology, Faculty of Medicine Nursing and Health Science, Monash University, Melbourne, Australia; 5 Department of Infectious Diseases, The Alfred hospital Melbourne, Melbourne, Australia; 6 Monash School of Medicine, Faculty of Medicine Nursing and Health Science, Monash University, Melbourne, Australia; 7 International Centre for Reproductive Health, Department of Obstetrics and Gynecology, Faculty of Medicine and Health Sciences, Ghent University, Ghent, Belgium

**Keywords:** Point-of-care testing, CD4, Pima, acceptability, feasibility, systematic review

## Abstract

**Background:**

CD4 testing is, and will remain an important part of HIV treatment and care in low and middle income countries (LMICs). We report the findings of a systematic review assessing acceptability and feasibility of POC CD4 testing in field settings.

**Methods:**

Electronic databases were searched for studies published in English between 2005 and 2015 that describe POC CD4 platforms. Studies conducted in LMICs and under field conditions outside a laboratory environment were eligible. Qualitative and descriptive data analysis was used to present the findings.

**Results:**

Twelve studies were included, 11 of which were conducted in sub-Saharan countries and used one POC CD4 test (The Alere Pima CD4). Patients reported positively regarding the implementation of POC CD4 testing at primary health care and community level with ≥90 % of patients accepting the test across various study settings. Health service providers expressed preference toward POC CD4 testing as it is easy-to-use, efficient and satisfied patients’ needs to a greater extent as compared to conventional methods. However, operational challenges including preference toward venous blood rather than finger-prick sampling, frequent device failures and operator errors, quality of training for test operators and supervisors, and increased staff workload were also identified.

**Conclusions:**

POC CD4 testing seems acceptable and feasible in LIMCs under field conditions. Further studies using different POC CD4 tests available on the market are required to provide critical data to support countries in selection and implementation of appropriate POC CD4 technologies.

**Electronic supplementary material:**

The online version of this article (doi:10.1186/s12913-016-1588-y) contains supplementary material, which is available to authorized users.

## Background

For many years, CD4 count testing has been a key diagnostic tool to identify HIV positive patients eligible for antiretroviral therapy (ART) and monitoring patient responses to treatment. Recently, in light of new evidence supporting early treatment [1], there is a movement in policy recommendation and practice towards CD4-independent ART initiation and a number of countries have already approved treatment for all HIV infected individuals regardless of their CD4 count. However, in the near future, CD4 counts still remain an important and practical part of HIV care particularly in decision making around ART initiation, clinical management and treatment monitoring in many countries where access to viral load monitoring remains limited [2].

Availability and access to CD4 count testing has been identified as a major barrier for increasing access to ART particularly in low-resource settings where laboratory based CD4 monitoring is not always available or easy to access [3]. The lack of reliable and affordable tests in these settings leads to missed opportunities of early ART initiation and significant patient loss-to-follow up [4].

Point-of-care (POC) CD4 testing has been introduced as an approach to overcome this challenge, possessing major advantages as compared to standard laboratory-based CD4 testing by flow cytometry in primary healthcare settings that lack infrastructure support and absence of well-functioning patient and/or sample referral systems [5]. Results from recent field studies suggest that POC CD4 tests are reliable [6–8], can help to increase the likelihood of an infected person having their CD4 T-cell count measured and receive the result [9], reduce time from HIV testing to ART initiation [10, 11], facilitate rapid (same day) ART initiation among patients eligible for treatment [12, 13], reduce loss to follow up [11], and most importantly, provide an immediate CD4 count which can significantly improve patient retention on care [14].

However, there is lack of synthesis of available evidence regarding operation and implementation of POC technologies in field settings. We conducted a systematic review to assess acceptability and feasibility of currently available or prototype commercial POC CD4 tests and to identify evidence gaps from field evaluations with a geographical focus in resource-constrained settings.

## Methods

### Literature search strategy

This review was conducted following the requirements of the preferred reporting items for systematic reviews and meta-analyses (PRISMA). A literature search using established search terms was first conducted in Medline to identify any study describing POC CD4 tests conducted in low and middle income countries (LMICs) published between Jan 2005 – Jan 2015 in English (see Additional file 1). The search strategy was then adapted by using the appropriate subject thesauri where available and modifying the search syntax to the relevant software platforms, and was undertaken across other electronic databases including: Embase, CENTRAL, Cinahl, PsycINFO, Biological Abstracts, Scopus and Web of Science. Searches were also conducted in grey literature resources and hand-searching of reference lists and citation was performed to identify relevant studies.

### Study selection

**Fig. 1:**
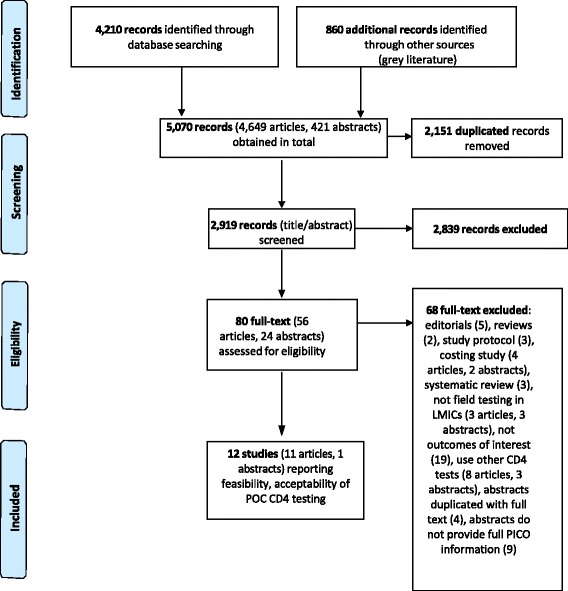
Selection process of included study for a systematic review of POC CD4 test

Study inclusion criteria were defined using PICO (participants, interventions, comparisons, outcomes) format [15]. Participants (P) included HIV positive, HIV negative and unknown HIV status persons aged ≥ 12 months. For intervention (I), any of the following six commercially available POC CD4 testing platforms listed in the UNITAID “2014 HIV/AIDS Diagnosis Technology Landscape” report [16] were included: (1) PointCare NOW™ (PointCare Technology Inc, Marlborough, MA, USA); (2) Pima™ CD4 (Alere Inc, Waltham, MA, USA); (3) Daktari™ CD4 Counter (Daktari Diagnostics Inc, Cambridge MA, USA); (4) CyFlow® CD4 miniPOC (Partec, Munich, Germany); (5) BD FACSPresto™ (BD Biosciences, San Jose, CA, USA); and (6) MyT4™ CD4 Test (Zyomyx Inc, Fremont, CA, USA). Comparators/controls (C): Laboratory based CD4 test (Flow Cytometry) if applicable with outcomes (O) of interest containing information on acceptability, feasibility of POC CD4 testing in field settings (Fig. 1).

### Data extraction and data synthesis

A pre-constructed electronic data extraction form was developed, pre-tested and finalized by consensus among authors. Data extraction was conducted by one reviewer and verified by the second using the data extraction form with 20 % duplicate extraction (Five papers were randomly selected and data extracted independently by 2 reviewers and compared to identify discrepancies if any). Quality of included studies was assessed using either the QUADAS tool for quality assessment of diagnostic studies included in systematic review [17], the EPHPP quality assessment tool for quantitative studies [18], or a specific tool for quality assessment of qualitative studies [19].

The following data items were extracted: author (s) and year of publication, intervention (name of POC CD4 technology, training for test operators where mentioned), comparison (if applicable), population (age, gender, HIV status), study setting (types of facility, location/country), study design and sample size, and key study outcomes of interest.

### Definitions and assessment of acceptability and feasibility

Acceptability and feasibility are closely linked and affect each other but they are not identical. Acceptability focuses on individual factors (provider and patient’s perspectives) while feasibility takes other system factors (infrastructure, human resource, policy) into account. Thus, one test could be acceptable to both provider and patient, but may not be feasible to be deployed in a specific setting (for example if a test device requires constant electricity supply or dedicated, well trained technicians to operate them). To assess acceptability and feasibility of POC CD4 testing in field settings, a conceptual framework initially developed to explore the feasibility, acceptance and use of a rapid diagnostic test for malaria [20] has been adapted for use in this study. From the service provider’s perspective, acceptability is assessed by the ability to understand how to correctly perform a POC CD4 test, their willingness to carry out the test when necessary as part of their daily work and their belief that the test is relevant to their work and test result is accurate. From patient’s perspective, acceptability is influenced by their willingness to have the test performed on themselves, their belief that the test is convenient to take and relevant in determining their CD4 count. Feasibility depends on acceptability and the presence or absence of supporting system factors such as training, monitoring/supervision, supplies, infrastructure such as space, light, water etc. that enable the implementation of POC CD4 testing in the field.

Based on this concept, the following pre-defined measurements were used to assess acceptability (1) uptake of POC CD4 testing: the proportion of individuals who accept a POC CD4 test (having blood samples drawn for POC CD4 test) of the total number of individuals who have been confirmed HIV positive and offered CD4 testing; (2) reported attributes of the POC CD4 test relating to day-to-day field operation from the relevant stakeholders’ perspectives (clients/patients, service providers, health manager/policy makers). For assessing feasibility the following measurements were used: (1) reported system factors associated with or having effect on acceptability and feasibility of POC CD4 test in the field, and (2) locally specific context and operational issues affecting the deployment of POC CD4 testing.

## Results

### Study characteristics

The search identified 12 studies that reported information and data on the outcomes of interest (acceptability and feasibility) including 11 articles and one abstract, 11 of which were conducted in Sub-Saharan countries and used Pima as a single intervention or as part of an intervention program to improve HIV testing uptake and linkage to care (Table 1). Overall, the quality of included studies was considered between moderate and strong. The QUADAS score ranged from 7 to 12 (of a maximum score of 14) with most of the studies scoring between 10 and 12. Using the EPHPP tool, two studies were ranked as “moderate” and one as “strong”. One study deployed both quantitative and qualitative methods, and addressed most of the essential criteria listed in the tool for appraising the quality of qualitative research.

**Table 1:** Characteristics of studies included in the review

Author (s), Year	Study objective	Study population/Study setting	Study design/Sample size	Sample/data collection	Intervention
Galiwango, 2014 [26]	To conduct a field evaluation assessing the accuracy of Pima	HIV infected patients (pre and experienced ART persons) at field clinics of Rakai Health Science Program in Rakai district, Southern rural Uganda. Study area has half a million population. Program is a community-based research organization with a focus on HIV/AIDS and reproductive health	Cross-sectional study A total of 903 patients were recruited among which 258 (28.5 %) patients were on ART	Venous blood samples collected by nursing team. Data was collected for clinical purpose and analyzed anonymously	Four Pima machines were used (machines were moved to testing site, located next to clinics, everyday from central Lab) Prior to daily testing, normal and low controls were run on each machine. Tests were run by qualified lab technicians who received Pima usage training
van Rooyen, 2013 [21]	To conduct assessment on effect of a home-based counseling and testing program that included POC CD4 testing on (1) high HIV testing coverage (2) identify newly infected or unaware HIV cases (3) reduce barriers to care and (4) increase access and adherence to ART	Known HIV-positive individuals older than 18 years in KwaZulu-Natal, South Africa. Study population characterized by high unemployment, low per capita income; and very high HIV prevalence (23.5 % among people aged ≤ 25). Study area was within walking distance of a primary health center and ART clinic	Prospective cohort study with one, three and six months follow-ups by lay counselors to evaluate outcomes 281 households enrolled, 671 adults consented and tested. Among 201 HIV infected participants, 193 had POC CD4 test in addition to CD4 lab-Cyflow.	POC CD4 testing was conducted at home using finger-prick blood sample. Venous blood samples were collected for BD FACSCalibur	Pima (as part of home-based counseling and testing program included POC CD4 testing, facilitated counseling and referrals) POC CD4 testing was conducted in the home at the same visit when positive HIV rapid test was obtained. POC test run by lay counselor/nurse assistant; training of test operators was not reported
Mtapuri-Zinyowera, 2013 [24]	To document experience in implementation of Pima in maternal and new-born child health setting in Zimbabwe	Clients (HIV positive women, lactating mothers, their families and other users) of health facilities with high-volume ANC visits of > 100 pregnant women seen/month located in 7 districts (five in each district) in Zimbabwe with and without Pima machines Key informants: relevant project staffs, MNCH staffs, counselors, lab staffs and ART personnel	Cross-sectional study 346 individuals were interviewed. Questionnaires were administered to 23 staff members, 62 trained POC users. Observation tools was applied to 22 trained users. Client exit questionnaire was administered to 142 clients of POC sites and 42 clients of non-POC sites. 1 client FGD conducted in each of 7 districts. Data of 207 client’s records was extracted from 45 facilities	Primary data was collected through face-to-face interviews, focus group discussion and observation using audio recorders and cameras (with verbal consent). Secondary data was extracted from medical records xxx	Pima Implemented at 35 ANC high-volume health facilities to provide CD4 count to HIV positive pregnant women and their families in hard to reach areas. Health care cadre and training of test operators not reported
Larson, 2012 [22]	To assess the impact of mobile HIV counseling and testing program on the proportion of patients completing referral visit within 8 weeks of HIV testing	Adult HIV positive patients diagnosed between May and November 2010 in a mobile HIV testing program (called ACCESS VCT) with 2 mobile units (with tents) to conduct HCT at sites (taxi rank, shopping mall) in Gauteng Province, South Africa	Retrospective cohort study A total of 508 patients were diagnosed with 311 patients were offered POC CD4 and 197 patients were not	Data was drawn retrospectively from routinely collected medical records kept by the ACCESS VCT program and completed in Feb 2011 allow for 8 weeks follow-up for all HIV positive patients	Four Pima devices were used in the same mobile location with each assigned to one nurse. With 6–10 nurses present during the day of testing patients were randomly assigned on a first-come first-serve basis; training of test operators not reported
Glencross, 2012 [31]	To report and compare the performance of Pima in laboratory or typical South African primary health HCT clinics	Adult HIV patients attending (1) Hospital based antenatal HCT clinic in Johannesburg-phase II (2) Two Primary health care HCT clinic in Limpopo province-phase IIIA; and (3) Inner-city primary health care clinic in Johannesburg, South Africa-phase IIIB	Cross-sectional study Phase II: *N* = 91 Phase IIIA: *N* = 96 Phase IIIB: *N* = 139	Both venous and capillary blood samples were collected	Pima operators (nursing personnel) were trained by the suppliers prior to commencing testing, according to methods defined by the manufacturer. Daily quality control was performed before commencing daily testing
Thakar, 2012 [25]	To assess the use of Pima at 21 ART centers in India	HIV positive patients aged 18–60 attending 21 ART centers in different parts of India having minimum (5-10/day) to moderate (25-30/day) patient load.	Cross-sectional study Total of 1790 participants were consecutively enrolled in 21 centers (5–10 HIV positive patients from each centre)	Both venous and capillary blood samples were collected	Technologists were trained for two days for finger prick sample collection & CD4 count estimation using Pima analyzer including the use of calibrators. Samples were run after low/normal control cartridge give acceptable values
Manabe, 2012 [27]	To evaluate performance of Pima in both laboratory and non-laboratory environment	HIV infected patients at Adult Infectious Diseases Institute Clinic within the Mulago Hospital Complex in Kampala, Uganda	Cross-sectional study *N* = 206.	Both venous and finger-prick blood samples were collected by study nurse	CD4 counts were performed using 4 Pima devices. Duplicate measurements were performed on both capillary and venous samples using 2 different devices. Test operator cadre and training not stated
Jani, 2011 [28]	To assess the ability of nurse to produce accurate results with POC test in primary health care settings providing ART	Documented HIV infected individuals from general patient population attending 2 primary health care setting providing a range of health services including ART in Maputo, Mozambique	Cross-sectional study *N* = 697.	Participants provided finger-prick (for POC tests) and venous blood (for lab-based tests)	Pima POC CD4 test operators were nurses in primary health clinics trained by the manufacturer. Manufacturer provided internal quality control and all POC instruments passed external qulity control assessment during study period
Mtapuri-Zinyowera, 2010 [23]	To evaluate the use of Pima and the ability of both nurses and laboratory technicians to run POC CD4 test	Newly diagnosed HIV positive patients at a VCT center at New Africa House in Harare, Zimbabwe	Cross-sectional study *N* = 165.	Participants provided finger-prick (for POC tests) and venous blood (for lab-based tests).	Two Pima devices were used. Nurses and laboratory technicians equally run POC CD4 tests (50/50) on each device. All test operators were formally trained on the Pima device and sample collection methodology for half a day
Wade, 2014 [30]	To assess performance and operational characteristics of Pima	HIV infected patients presenting for routine CD4 testing at infectious disease clinic in Dar es Salam (Tanzania)	Cross-sectional study *N* = 200.	Both capillary blood (Pima) and venous blood (FACSCalibur) were collected	Pima test operator cadre and training not reported. Pima testing procedures were not described
Mwau, 2014 [7]	To evaluate the technical performance of MyT4 POC CD4 test	HIV infected patients ≥ 18 years old at comprehensive HIV care clinics of 2 health care facilities in Busia county of Western province, Kenya	Cross-sectional study *N* = 276.	Finger-prick blood samples (for MyT4 test) and venous blood samples for conventional CD4 tests collected.	All samples were collected and tested using MyT4 POC CD4 by trained health care staffs (nurses and lab technicians) Training for staff not reported
Arnett N, 2013 [29]	To assess healthcare worker acceptance and ability to perform POC CD4 test	HIV infected patients from 5 PMTCT and HIV treatment sites in Dar-es-Salaam, Tanzania	Cross-sectional study 1060 patients provided blood specimens, 11 HCWs interviewed	Each participant provided 3 samples: (1) venous (1) finger-prick directly to PIMA cartridge and (1) finger-prick collected into Microtube	Pima POC CD4 tests run by trained healthcare workers

HCT: HIV Counseling and Testing; ANC: Antenatal clinic; MNCH: Maternal and new-born child health; ART: Antiretroviral therapy; PMTCT: Prevention of mother to child transmission

### Acceptability and feasibility of POC CD4 test

Only one of the included studies, presented as a conference abstract, aimed to assess acceptability of a POC CD4 test from the healthcare workers’ perspective. All other studies primarily assessed accuracy and/or effect of POC CD4 on HIV program or patient related outcomes, and provided additional data related to acceptability and feasibility of POC CD4 testing under field conditions (Table 2).

**Table 2:** Acceptability and feasibility of POC CD4 test

Author (s), Year	Technologies	Proportion of HIV patients accepted POC CD4 test when offered	Reported attributes of POC CD4 test related to day-to-day field operation	System Factors associated with/having effect on acceptability/feasibility of POC CD4 test	Locally specific context and operational issues which affect the deployment of POC CD4 test
Galiwango, 2014 [26]	Pima		Easy to use; enable same day, on-site immunological assessment and result communication		In busy clinic, it requires 2–4 machines with additional technician to complete patient testing
van Rooyen, 2013 [21]	Pima	Highly acceptable at the time of learning about HIV test result (96 % of identified HIV positive individuals accepted, tested and received POC CD4 count result at a HBCT visit	Feasible to be conducted at homes, as part of home-based HIV counseling and testing program, in a rural South African setting		
Mtapuri-Zinyowera, 2013 [24]	Pima		Relatively low throughput, frequent error codes and cartridge rejection before expiration date. Increased technical breakdown after 1 year of operation at busy sites; major breakdowns include hardware and alignment and loss of camera focus 25 % of users reported having some challenges after machine installation of which 67 % (of cases) were resolved by the Manufacturer and 33 % by users	Users reported training was useful and relevant to day-to-day operation. Training for supervisor is needed to monitor staff performance. External quality control was a challenge because of remoteness of sites Challenges with finger-prick sampling is also noted including that it cannot be used for full blood count (required to determine types of ARVs patient can take), high error rate led to multiple finger-prick exposing patient to more pain	Staff workload was the most prominent challenge reported by users (multiple tasks and increased workload without compensation); task shifting should be considered given prospect of additional staff employment is low
Larson, 2012 [22]	Pima	When offered a rapid POC CD4 test in a routine mobile HCT setting, acceptance among patients is high (90 %); only 32 /311 (10.3 %) patients declined the offer of POC CD4			
Glencross, 2012 [31]	Pima			Negative impact of (poor) capillary blood sampling on POC CD4 test performance: Capillary sampling demands absolute diligence and stringency of sampling technique. Ongoing dedicated training as well as implementation of systems for monitoring and evaluation of testing is strongly recommended	
Thakar, 2012 [25]	Pima		Users expressed that PIMA was compact and hence could fit in the small space available at the centers. It is battery operated, showed a battery backup of 3–4 h eliminating requirement of continuous electricity	Study participants preferred to give venous blood sample because of requirement of blood collection for other investigations using venous blood and a fear of being subjected to multiple pricks if sufficient volume of blood is not obtained in a single prick	
Manabe, 2012 [27]	Pima		Easy-to-use, portable, relatively fast device to test CD4+ T cell counts in the field	Quality control and observed practical training for test operators would be required to ensure that good volume and flow of blood (capillary) is obtained	
Jani, 2011 [28]	Pima		Essential WHO-recommended ART staging and monitoring diagnostic tests can be accurately conducted at primary health care clinic level by non-laboratory staff using POC CD4 test	Operators should be trained for finger prick testing and their performance should be regularly monitored as training and monitoring has been shown to be essential to the ongoing reliability of other POC CD4 test	Implementation of POC CD4 in primary health care clinics requires careful planning. Task shifting of ART services to community clinics places additional strain on the workloads of nurses and other healthcare workers that may be unsustainable
Mtapuri-Zinyowera, 2010 [23]	Pima	The offer of POC CD4 testing within post-test counseling was accepted by almost all eligible clients, even within the context of a study and the need to provide informed consent.	POC CD4 testing can be performed in non-laboratory setting by non-laboratory technicians (nurses)	It is important to ensure that Pima test operators are well trained on finger-prick sample collection. Preliminary observations in this study suggest that incorrect finger-prick sampling affects the reliability of POC CD4 results	
Wade, 2014 [30]	Pima			Significant contribution of operators to variability of POC CD4 test results: dedicated training for test operators, particularly on capillary blood sampling is required to ensure quality of POC CD4	
Mwau, 2014 [7]	MyT4		Relatively high throughput: over 20 tests/6 h health facility working day		Implementation would be most effective by assigning a dedicated full time operator
Arnet N, 2013 [29]	Pima		100 % (11/11) HCW interviewed trust Pima venous CD4 results; 91 % (10/11) for Pima Microtube and 82 % (9/11) for Pima direct. The most preferred sample collection method was Pima venous 73 % (8/11)		

*HBCT* home-based HIV counseling and testing

### Patient perspectives

Available data suggest that the Pima test was highly acceptable across study settings. Acceptance rates ranged from 90 % to 100 % when offered either at the home, via mobile HIV counseling and testing or in permanent voluntary counseling and testing settings [21–23]. Patients appreciated having on-site CD4 testing availability. One participant reported: “They used to collect our blood and send it somewhere, hence that will take some days but as for now there is a big difference, we now receive our results there and then” [24]. In terms of preference regarding the method of blood sampling, the findings of one study in India [25] suggested that study participants prefer to give venous blood samples versus finger-prick because of the requirement for other blood tests (which can only be performed with venous blood) and fear of being subjected to the discomfort of multiple pricks with more pain if sufficient volume is not obtained in one prick.

### Provider perspectives

One study which employed qualitative research methods in 35 maternal and new-born child health (MNCH) settings in Zimbabwe [24] reported health personnel expressed a preference toward performing POC CD4 testing (Pima) as it was efficient in resource use, user-friendly and responded well to patients’ needs: the machine “used fewer resources (syringes and needles), and less technical expertise was needed” to run the test and it was “catering for all types of clients”; finger-prick sampling was reported as “less traumatizing to the patients as less blood is taken for immediate use” and “no suspicion that blood was used for other purposes”. The Pima machine was reported as portable, compact, easy-to-use, battery operated with 3–4 h backup providing feasibility for use in non-laboratory settings [21, 25–27] and by non-laboratory technicians [23, 28]. However, relatively low throughput capacity, frequent error codes, cartridge rejection before expiration date and increased technical breakdown after one year of operation at a busy site were also reported as limitations of the Pima technology [24]. Healthcare workers also noted that a full blood count is required to identify type of antiretroviral drugs patients can be prescribed and this cannot be done with a finger-prick sample; also problems with test errors may lead to multiple pricks being required [24]. In another study conducted at five Prevention of Mother to Child Transmission (PMTCT) and HIV/AIDS care and treatment clinics in Tanzania, 73 % (8/11) of health care workers who were interviewed named venous blood as their preferred sample collection method and 100 % (11/11) trusted Pima-venous test results [29].

### Influencing factors

Human resource shortage is a major factor that influences feasibility and acceptability of POC CD4 in field settings. It was suggested that the introduction of onsite POC CD4 testing would increase the workload of health personnel at clinical settings and this could be considered the most prominent challenge in terms of feasibility and acceptability in the context of no financial incentives or additional staffing could be provided [24, 28]. Training for health care staff on POC CD4 testing was reported as another factor that might influence acceptability and feasibility. In addition to training of test operators, training for health managers is also required. Senior health staff reported that it was not feasible for them to monitor test operators on a test on which they were not trained: “it’s difficult for me to supervise something I don’t know about” and “it’s downgrading to received instruction from a junior” [24]. The internal quality control and performance monitoring were also reported as critical factors to ensure on-going reliability and acceptability of POC CD4 testing in clinic settings where external quality control is a challenge because of the remoteness of the sites [27, 30, 31].

## Discussion

The findings of our review show that there is relatively limited data on acceptability and feasibility of POC CD4 testing, with data only available for the Pima test; no other current commercially available POC CD4 tests [32] have published data on acceptability and feasibility in field settings. For Pima, the available data consistently demonstrates that it is feasible to deploy for decentralization of CD4 testing through different models of service delivery. However, there are some issues related to the implementation of Pima that might influence acceptability and feasibility of the test that remain unanswered. From the patient’s perspective no qualitative data were available to capture the willingness of patients to choose a POC CD4 test over a standard flow cytometric CD4 test when offered, or the patient’s belief in the accuracy or relevance of POC CD4 testing in identifying treatment eligibility or response to treatment. One study [22] reported 10 % of patients declined the offer of POC CD4 testing and chose standard CD4 testing at referral clinics but reasons for their preference was not studied. From the health service provider’s perspective, a number of issues need to be addressed in terms of acceptability and feasibility to provide insights into future implementation of the test. Although the device was reported as being easy-to-use, a reported high error rate and frequent test operator errors raised questions about the health workers’ ability to correctly perform the test in their daily working environment. Information on the training and supervision of test operators that would allow assessment of their capacity to follow testing procedure and perform the test correctly is lacking. The preference of health workers toward venous blood collection for CD4 testing also needs further studies to identify if this preference is due to the health workers’ belief in the validity of the test, difficulties involved with capillary blood sampling or other logistic issues related to blood testing in field settings. Lastly, human resources shortage represents a major challenge. The introduction of POC CD4 testing at primary healthcare level has been reported to be associated with increased work-load for health staff and while the provision of this service may well response to patients’ needs and improve quality of HIV treatment and care, the question remains is how to provide effective and sustainable POC CD4 testing service in busy primary clinic settings without overburdening the already strained healthcare providers. All these are important issues that need to be studied and factored into the planning process to guide future implementation of the test in remote and disadvantaged areas.

Although data was only available for Pima POC CD4 testing, some lessons could be learned for future planning and implementation of POC CD4 testing in LMICs. First, like other rapid diagnostic tests the validity or accuracy of POC CD4 test as perceived by the end users (health care provider and patient), will affect acceptance and use of the test in the field [20]. As accuracy and efficacy of the test are influenced by the test operators’ practical skills, the importance of quality training on POC CD4 testing for health professionals, both test operator and supervisor, must be recognized and infrastructure to support this should be adequately addressed before introduction and scale up of any POC CD4 technology [33]. This remains true for all POC tests that could be considered “quick” and “easy” but often require comprehensive training and supervision to ensure diagnostic test accuracy under field conditions [34, 35]. Without standardized quality training packages delivered to health workers in these clinics, ensuring competency standards are obtained by all test operators and their supervisors, and without on-going internal quality assurance systems in place it is impossible to rule out any potential operator-induced bias. Second, the preference toward a specific type of blood sample may also influence the acceptability and feasibility of a POC CD4 test. This preference may come from either the health service provider’s or patient’s views including challenges with one type of blood sampling, the need for a large amount of blood for other blood tests, or the health worker’s belief in accuracy of the test with one type of blood over the other. For POC CD4 tests that can be used with either type of blood sample, this would require further study to identify technical and programmatic solutions for extending test applicability in the field. Third, POC technologies are designed to be user friendly and be easily operated by low-to-middle level healthcare cadres. In addition to the importance of training for health staff, discussed above, their workload upon introduction of POC CD4 testing is an important issue to consider. The reality is that in some of the highest volume clinics in low-resource settings, it is the nurses, midwives and counselors who have the highest workload. Therefore, it is recommended that patient and staff work flow should be thoroughly studied and work load issues appropriately addressed through additional incentives, staffing or task shifting to ensure the effectiveness, efficacy and sustainability of POC technology when introduced into busy clinical settings.

The future role and impact of CD4 testing in general and POC CD4 testing in particular need to be considered from both technical and programmatic perspectives. In order to achieve the newly proposed sustainable development goal of ending the HIV/AIDS epidemic by 2030 [36], and with evidence showing positive clinical impact of ART for patients with CD4 count > 500 cells/μl [37], a policy shift toward recommendation of ART initiation for all HIV infected individuals regardless of CD4 count has recently been announced by WHO [38]. However, scaling up of ART programs in low-resource settings may risk low adherence and retention to care rates if critical health system factors such as well-trained health staff, well-functioning patient monitoring and appropriate support systems are not in place [39]. A successfully scaled up treatment program, therefore, will require innovative and effective models of service delivery (such as integration and decentralization of care), strong procurement and supply chain management, sufficient laboratory and/or point-of-care diagnostic services to monitor clinical treatment outcomes, HIV viral load and drug resistance and antiretroviral drug toxicities. Most of these systems are not currently in place in those LMICs mostly affected by the HIV epidemic and could only be achieved through a gradual process of health system strengthening. Therefore, in countries where treatment for all is not currently feasible with the available resources and current health system capacity, CD4 testing is required to prioritize treatment eligibility likely for those patients whose CD4 count is less than 350 cells/μl. Going forward, CD4 testing will still play an important role in identifying patients who present late to care for clinical management, for initiation and cessation of prophylaxis and for management of patient responses to ART even in countries where CD4-independent ART initiation is being introduced.

This review has some limitations that should be considered in interpretation of the findings. First, assessment of acceptability and feasibility was not the primary objective of any of the included studies and in a number of studies POC CD4 testing was only a part of a more comprehensive program intervention. This carries a risk of introducing bias as impact of the POC CD4 test and its acceptability and feasibility could be influenced by other interventions or implementation strategies. Second, we included only published studies in English and this inclusion may overlook data from studies published in other languages or unpublished data from evaluations conducted by government agencies, local reference facilities or research institutions. All but one of the included studies reporting outcomes of interest use Pima as the index test and this presents challenges in terms of generalizing the findings of the review to other POC CD4 tests, as specific technical and operational characteristics of each test will significantly impact feasibility and acceptability of the test from both service provider and patient perspectives. Of note, these limitations cannot be overcome until more data from field studies of different POC CD4 technologies are available.

## Conclusions

Data is available for one of the POC CD4 tests on the market, the Alere Pima CD4, and suggests that it could be feasible to implement point-of-care CD4 testing in non-laboratory settings in low and middle income countries. Further studies using other currently or newly available POC CD4 tests in different geographical regions are needed to inform in-country decision making regarding the selection and adoption of a suitable test. Qualitative studies on feasibility and acceptability are needed to explore end users’ beliefs on the value of and preference toward POC CD4 testing. Evidence regarding supporting system factors such as training, monitoring, supplies, and facility requirements should be available and inform the planning process for the introduction and scaling up of POC CD4 tests at primary health care levels in low-resource settings.

## Abbreviations

ART, Antiretroviral therapy; HBCT, home based counseling and testing; LMICs, Low and middle income countries; LTFU, Loss to follow up; MNCH, Maternal and new born child health; PICO, participants, interventions, compactors/controls, outcomes; POC, point-of-care; PRISMA, Preferred reporting items for systematic review and meta-analysis

## Additional file

Additional file 1: Medline search strategy (DOC 50 kb)

## References

[1] INSIGHTSTART (2015). Initiation of Antiretroviral Therapy in Early Asymptomatic HIV Infection. N Engl J Med.

[2] Ford N (2015). The future role of CD4 cell count for monitoring antiretroviral therapy. Lancet Infect Dis.

[3] Zachariah R (2011). Viewpoint: Why do we need a point-of-care CD4 test for low-income countries?. Trop Med Int Health.

[4] Govindasamy D, Ford N, Kranzer K (2012). Risk factors, barriers and facilitators for linkage to antiretroviral therapy care: a systematic review. AIDS.

[5] Reid S, Fidler S, Cooke G (2013). Tracking the progress of HIV: The impact of point-of-care tests on antiretroviral therapy. Clin Epidemiol.

[6] Rathunde L (2014). Evaluation of the Alere Pima™ for CD4+ T lymphocytes counts in HIV-positive outpatients in Southern Brazi. Int J STD AIDS.

[7] Mwau M (2014). Technical Performance Evaluation of the MyT4 Point of Care Technology for CD4+ T Cell Enumeration. PLoS ONE.

[8] Scott LE (2015). A meta-analysis of the performance of the Pima (TM) CD4 for point of care testing. BMC Med.

[9] Wynberg E (2014). Impact of point-of-care CD4 testing on linkage to HIV care: a systematic review. J Int AIDS Soc.

[10] Patten GE (2013). Impact on ART initiation of point-of-care CD4 testing at HIV diagnosis among HIV-positive youth in Khayelitsha, South Africa. J Int AIDS Soc.

[11] Jani IV (2011). Effect of point-of-care CD4 cell count tests on retention of patients and rates of antiretroviral therapy initiation in primary health clinics: an observational cohort study. Lancet.

[12] Black S (2014). Acceptability and challenges of rapid ART initiation among pregnant women in a pilot programme, Cape Town, South Africa. AIDS Care.

[13] Myer L (2012). Pilot programme for the rapid initiation of antiretroviral therapy in pregnancy in Cape Town, South Africa. AIDS Care.

[14] Faal M (2011). Providing immediate CD4 count results at HIV testing improves ART initiation. J Acquir Immune Defic Syndr.

[15] Counsell C (1997). Formulating questions and locating primary studies for inclusion in systematic reviews. Ann Intern Med.

[16] UNITAID (2014). 2014 HIV/AIDS Diagnostics Technology Landscape.

[17] Whiting P (2003). The development of QUADAS: a tool for the quality assessment of studies of diagnostic accuracy included in systematic reviews. BMC Med Res Methodol.

[18] Jackson N (2005). Criteria for the systematic review of health promotion and public health interventions. Health Promot Int.

[19] Walsh D, Downe S (2006). Appraising the quality of qualitative research. Midwifery.

[20] Asiimwe C (2012). Early experiences on the feasibility, acceptability, and use of malaria rapid diagnostic tests at peripheral health centres in Uganda-insights into some barriers and facilitators. Implement Sci.

[21] Van Rooyen H (2013). High HIV testing uptake and linkage to care in a novel program of home-based HIV counseling and testing with facilitated referral in KwaZulu-Natal, South Africa. J Acquir Immune Defic Syndr.

[22] Larson BA (2012). Rapid point-of-care CD4 testing at mobile HIV testing sites to increase linkage to care: an evaluation of a pilot program in South Africa. J Acquir Immune Defic Syndr.

[23] Mtapuri-Zinyowera S (2010). Evaluation of the PIMA point-of-care CD4 analyzer in VCT clinics in Zimbabwe. J Acquir Immune Defic Syndr.

[24] Mtapuri-Zinyowera S (2013). PIMA Point of Care CD4+ Cell Count Machines in Remote MNCH Settings: Lessons Learned from Seven Districts in Zimbabwe. Infect Dis (Auckl).

[25] Thakar M (2012). Utility of the point of care CD4 analyzer, PIMA, to enumerate CD4 counts in the field settings in India. AIDS Res Ther.

[26] Galiwango RM (2014). Field evaluation of PIMA point-of-care CD4 testing in Rakai, Uganda. PLoS ONE.

[27] Manabe YC (2012). Evaluation of portable point-of-care CD4 counter with high sensitivity for detecting patients eligible for antiretroviral therapy. PLoS ONE.

[28] Jani IV (2011). Accurate CD4 T-cell enumeration and antiretroviral drug toxicity monitoring in primary healthcare clinics using point-of-care testing. AIDS.

[29] Arnett N, Chang K, Schmitz M, Lemwayi R, Rwehumbiza P, Mwasekaga M (2013). Healthcare workers’ acceptance and performance of point-of-care CD4 testing in Dar es Salaam, Tanzania, 2011.

[30] Wade D (2014). WHO multicenter evaluation of FACSCount CD4 and Pima CD4 T-cell count systems: instrument performance and misclassification of HIV-infected patients. J Acquir Immune Defic Syndr.

[31] Glencross DK (2012). Performance evaluation of the Pima™ point-of-care CD4 analyser using capillary blood sampling in field tests in South Africa. J Int AIDS Soc.

[32] UNAIDS (2015). HIV/AIDS Diagnostics technologies Landscape Semi-Annual Update.

[33] Fajardo E (2015). Errors generated by a point-of-care CD4+ T-lymphocyte analyser: a retrospective observational study in nine countries. Bull World Health Organ.

[34] Seidahmed OM (2008). End-user errors in applying two malaria rapid diagnostic tests in a remote area of Sudan. Trop Med Int Health.

[35] Wolpaw BJ (2010). The failure of routine rapid HIV testing: a case study of improving low sensitivity in the field. BMC Health Serv Res.

[36] Nations, U (2015). Final draft of the outcome document for the UN Summit to adopt the Post-2015 Development Agenda.

[37] TEMPRANO (2015). A Trial of Early Antiretrovirals and Isoniazid Preventive Therapy in Africa. N Engl J Med.

[38] WHO (2015). GUIDELINE ON WHEN TO START ANTIRETROVIRAL THERAPY AND ON PRE-EXPOSURE PROPHYLAXIS FOR HIV.

[39] Unge C (2009). Challenges for scaling up ART in a resource-limited setting: a retrospective study in Kibera, Kenya. J Acquir Immune Defic Syndr.

