# Supplementary material for: Acceptability and feasibility of point-of-care CD4 testing on HIV continuum of care in low and middle income countries: a systematic review
Source: BMC Health Serv Res. 2016 Aug 2;16:343. doi: 10.1186/s12913-016-1588-y (PMC4971709; doi:10.1186/s12913-016-1588-y)
Supplement: Additional file 1: — Medline search strategy (DOC 50 kb) [file 12913_2016_1588_MOESM1_ESM.doc]

**Additional file 1: Medline Search Strategy**

Database(s): **Ovid MEDLINE(R)**1946 to January Week 2 2015

| **#** | **Searches** |
| --- | --- |
| 1 | exp CD4 Lymphocyte Count/ or exp CD4-Positive T-Lymphocytes/ or Antigens, CD4/ |
| 2 | CD4.tw. |
| 3 | (T4 adj3 (cell* or lymphocyte*)).tw. |
| 4 | 1 or 2 or 3 |
| 5 | Point-of-Care Systems/ or early diagnosis/ or Mobile Health Units/ |
| 6 | (point-of-care or point-of-patient-care or point-of-service or point-of-need or site-of-patient-care).tw. |
| 7 | (POC or POCT).tw. |
| 8 | (PIMA or PointCare or miniPOC or Cyflow or Daktari or FACSPresto or MyT4).mp. |
| 9 | ((rapid or immediate or prompt or mobile or portable or early) adj4 (test* or assay*)).tw. |
| 10 | ((rapid or immediate or prompt or mobile or portable or early) adj2 (count* or enumerat* or result*1)).tw. |
| 11 | 5 or 6 or 7 or 8 or 9 or 10 |
| 12 | 4 and 11 |
| 13 | limit 12 to (english language and yr="2005 -Current") |
| 14 | Antiretroviral Therapy, Highly Active/ or exp Anti-Retroviral Agents/ or exp Anti-hiv Agents/ |
| 15 | (HAART or ART).tw. |
| 16 | (anti-retroviral or antiretroviral or anti-HIV or antiHIV).tw. |
| 17 | 14 or 15 or 16 |
| 18 | 11 and 17 |
| 19 | Developing Countries/ |
| 20 | (Afghanistan* or Albania* or Algeria* or Angola* or Argentina* or Armenia* or Azerbaijan* or Bangladesh* or Belarus* or Beliz* or Benin* or Bhutan* or Bolivia* or Bosnia* or Herzegovin* or Botswan* or Brazil* or Bulgaria* or Burkina* or Burundi* or Cabo Verde* or Cape Verde* or Cambodia* or Cameroon* or Central African or Chad* or China or Chinese or Colombia* or Comor* or Congo* or Costa Rica* or Cote d'Ivoir* or Ivory Coast or Cuba* or Djibouti* or Dominica* or Ecuador* or Egypt* or El Salvador* or Eritrea* or Ethiopia* or Fiji* or Gabon* or Gambia* or Georgia* or Ghana* or Grenad* or Guatemala* or Guinea* or Guyan* or Haiti* or Hondura* or Hungar* or India* or Indonesia* or Iran* or Iraq* or Jamaica* or Jordan* or Kazakhstan* or Kenya* or Kiribati* or Korea* or Kosov* or Kyrgyz Republic or Lao* or Leban* or Lesotho* or Liberia* or Libya* or Macedonia* or Madagascar* or Malawi* or Malaysia* or Maldiv* or Mali* or Marshall Island* or Mauritania* or Mauriti* or Mexic* or Micronesia* or Moldova* or Mongolia* or Montenegr* or Morocc* or Mozambi* or Myanma* or Burmese or Namibia* or Nepal* or Nicaragua* or Niger* or Nigeria* or Pakistan* or Palau* or Panama* or Papua New Guinea* or Paraguay* or Peru* or Philippines or Filipino or Romania* or Rwanda* or Samoa* or Sao Tome* or Senegal* or Serbia* or Seychell* or Sierra Leon* or Solomon Island* or Somalia* or South Africa* or Sudan* or Sri Lanka* or St Lucia* or St Vincent or Grenadines or Surinam* or Swazi* or Syria* or Tajikistan* or Tanzania* or Thai* or Timor* or Togo* or Tonga* or Tunisia* or Turk* or Turkmenistan* or Tuvalu* or Uganda* or Ukrain* or Uzbekistan* or Vanuatu* or Venezuela* or Vietnam* or West Bank or Gaza or Yemen* or Zambia* or Zimbabwe*).mp. |
| 21 | exp africa/ or exp caribbean region/ or exp central america/ or latin america/ or exp south america/ or asia/ or exp asia, central/ or exp asia, southeastern/ or exp asia, western/ or exp indian ocean islands/ or pacific islands/ or exp melanesia/ or exp micronesia/ or exp west indies/ |
| 22 | (africa* or asia* or caribbean or central america* or latin america* or south america* or melanesia* or micronesia* or polynesia*).tw. |
| 23 | (resource-limit* or resource-poor or low-resource* or limited-resource* or resource-constrain* or constrain*-resource* or under-resource* or poor*-resource* or resource-scarce* or scarce*-resource* or low-income or middle-income or lowincome or middleincome or LMIC*).tw. |
| 24 | ((developing or underdeveloped or under-developed or emerging or less-developed or least-developed or less-economically developed or least-economically developed or less-affluent or least-affluent) adj (country or countries or nation or nations or region or regions or economy or economies)).tw. |
| 25 | ((developing or underdeveloped or under-developed or less-developed or least-developed) adj world).tw. |
| 26 | (third-world* or thirdworld* or 3rd-world*).tw. |
| 27 | 19 or 20 or 21 or 22 or 23 or 24 or 25 or 26 |
| 28 | 18 and 27 |
| 29 | limit 28 to (english language and yr="2005 -Current") |
| 30 | 13 or 29 |
| 31 | exp animals/ not humans.sh. |
| 32 | 30 not 31 |
